# The feasibility of a randomized controlled trial of esophagectomy for esophageal cancer - the ROMIO (Randomized Oesophagectomy: Minimally Invasive or Open) study: protocol for a randomized controlled trial

**DOI:** 10.1186/1745-6215-15-200

**Published:** 2014-06-02

**Authors:** Kerry NL Avery, Chris Metcalfe, Richard Berrisford, C Paul Barham, Jenny L Donovan, Jackie Elliott, Stephen J Falk, Rob Goldin, George Hanna, Andrew A Hollowood, Richard Krysztopik, Sian Noble, Grant Sanders, Christopher G Streets, Dan R Titcomb, Tim Wheatley, Jane M Blazeby

**Affiliations:** 1 School of Social and Community Medicine, University of Bristol, 39 Whatley Road, BS8 2PS, Clifton Bristol, UK; 2 Department of Upper Gastrointestinal Surgery, Derriford Hospital, Derriford Road, PL6 8DH Plymouth, UK; 3 Division of Surgery, Head and Neck, University Hospitals Bristol NHS Foundation Trust, Bristol Royal Infirmary, Marlborough Street, BS1 3NU Bristol, UK; 4 Gastro-Oesophageal Support and Help Group, 15 Honey Hill Road, BS15 4HG Kingswood, South Gloucestershire, UK; 5 Bristol Oncology Centre, University Hospitals Bristol NHS Foundation Trust, Horfield Road, BS2 8ED Bristol, UK; 6 Centre for Pathology, 4th Floor Clarence Wing, St. Mary’s Hospital, Praed Street, W2 1NY London, UK; 7 Department of Bio-Surgery & Surgical Technology, Imperial College NHS Trust, Academic Surgical Unit, 10th Floor, QEQM Building, St. Mary’s Hospital, Praed Street, W2 1NY London, UK; 8 Gastroenterology & Surgical Department B57, Royal United Hospital Bath NHS Trust, Combe Park, BA1 3NG Bath, UK

**Keywords:** Feasibility studies, Upper gastrointestinal neoplasms, Pilot study, Surgical procedures, Minimally invasive, Randomized controlled trial

## Abstract

**Background:**

There is a need for evidence of the clinical effectiveness of minimally invasive surgery for the treatment of esophageal cancer, but randomized controlled trials in surgery are often difficult to conduct. The ROMIO (Randomized Open or Minimally Invasive Oesophagectomy) study will establish the feasibility of a main trial which will examine the clinical and cost-effectiveness of minimally invasive and open surgical procedures for the treatment of esophageal cancer.

**Methods/Design:**

A pilot randomized controlled trial (RCT), in two centers (University Hospitals Bristol NHS Foundation Trust and Plymouth Hospitals NHS Trust) will examine numbers of incident and eligible patients who consent to participate in the ROMIO study. Interventions will include esophagectomy by: (1) open gastric mobilization and right thoracotomy, (2) laparoscopic gastric mobilization and right thoracotomy, and (3) totally minimally invasive surgery (in the Bristol center only). The primary outcomes of the feasibility study will be measures of recruitment, successful development of methods to monitor quality of surgery and fidelity to a surgical protocol, and development of a core outcome set to evaluate esophageal cancer surgery. The study will test patient-reported outcomes measures to assess recovery, methods to blind participants, assessments of surgical morbidity, and methods to capture cost and resource use. ROMIO will integrate methods to monitor and improve recruitment using audio recordings of consultations between recruiting surgeons, nurses, and patients to provide feedback for recruiting staff.

**Discussion:**

The ROMIO study aims to establish efficient methods to undertake a main trial of minimally invasive surgery versus open surgery for esophageal cancer.

**Trial registration:**

The pilot trial has Current Controlled Trials registration number ISRCTN59036820(25/02/2013) at http://www.controlled-trials.com; the ROMIO trial record at that site gives a link to the original version of the study protocol.

## Background

Esophageal cancer is the ninth most common cancer in the United Kingdom (UK), and around 8,000 people are newly diagnosed with the disease each year [1]. Surgery alone or in combination with chemotherapy or chemoradiation treatment is the mainstay of cure for localised esophageal adenocarcinoma and one of several options for esophageal squamous cell cancer, which may also be radically treated with definitive chemoradiotherapy or radiotherapy alone. Esophagectomy for esophageal cancer is a major procedure. Audit data for 1220 esophagectomies carried out in England and Wales from April 2011 to March 2012 showed that 29.7% of patients would experience a complication while 8.9% would experience serious morbidity requiring a re-operation [2]. The thirty-day mortality rate is 1.7% [2]. Esophagectomy also has a major short-term detrimental impact on health-related quality of life (HRQL), with patients reporting a reduction in physical and social function and marked increases in fatigue, breathlessness, and pain scores for at least three months after surgery [3,4] and persistent long-term deficits can occur [3]. These morbidities need to be considered alongside the evidence that esophagectomy offers long-term survival in the region of 20 to 40% [5-7].

There are several approaches for the resection of esophageal tumors. Surgery may involve two or three phase procedures (abdominal, chest and/or neck incisions) or a transhiatal approach (abdominal and cervical incision); each of these may be performed with minimal access or open approaches. The past decade has seen a growing interest in minimal access surgical techniques for all types of cancer surgery, with the potential advantages of causing less tissue trauma and better recovery. In England and Wales, 43% (492 out of 1140) of esophagectomies performed during the period of the recent national audit used minimal access surgical techniques [2]. These were mostly (321 out of 492, 65%) laparoscopically assisted two-phase approaches (minimal access approach for the abdomen and standard open right chest incision), with the others being totally minimally invasive esophagectomy (MIO). The audit data suggested that length of hospital stay and inpatient complication rates following open and minimal access approaches were similar, with the small differences observed being consistent with chance. The biggest difference was in respiratory complications, the rate of which was observed to be lower in MIO (11%, 18 out of 171 assuming no missing data) than in open procedures (17%, 108 out of 647), a difference which is unlikely to occur by chance (*P* = 0.047, chi-square test), but the interpretation of this finding is complicated by the highest rate of respiratory complications occurring following the hybrid procedures (22% 70 out of 321). This is one example of the complex data that can result from observational studies, where the different procedures may be undertaken by surgeons with different levels of experience and in patients selected for particular interventions with different baseline prognostic factor (for example age, fitness, and tumor stage) profiles.

Whilst a number of systematic reviews have been conducted, these all focus on data from observational studies [8-17]. Such studies are subject to the same caveats as stated above, and whilst they suggest that minimally invasive techniques may improve short-term clinical outcomes (such as morbidity and physiological measures) [18] and reduce impact on HRQL during recovery [19], these results must be interpreted with caution. Furthermore, even these weak studies tell us little about long-term survival [20], cost effectiveness, and impact on long-term HRQL [8,21].

There are a limited number of small randomized trials, including the recently reported Dutch TIME trial (Traditional invasive versus minimally invasive esophagectomy), which compared open two or three phase esophagectomy (56 patients) with totally MIO (59 patients) and reported short-term outcome data [22]. The trial provided evidence that minimally invasive esophagectomy was associated with a lower incidence of in-hospital pulmonary infection (12% versus 34%) and a shorter hospital stay (median 11 days versus 14 days) compared to open surgery. The trial report was followed by a critical correspondence from the surgical community [23-26], much of which reflected a failure to appreciate the pragmatic approach necessary to evaluate complex interventions [27]. At the time of writing, the French MIRO (oesophagectoMIe pour cancer paR voie conventionnelle ou coeliO-assistée) trial is in progress, randomly allocating 200 patients between open and laparoscopically-assisted esophagectomy. Again the focus will be on short-term complications (major morbidity within 30 postoperative days) [28]. Whilst both the TIME and MIRO trial contribute important data, there is a need for a larger multicenter trial that is designed to assess the longer-term clinical effectiveness of minimally invasive surgery. This trial needs to have a sufficient sample size to demonstrate that any advantages of the minimally invasive procedure for recovery and longer-term HRQL are achieved without compromising the survival benefits of surgical intervention.

There are a number of challenges to conducting randomized controlled trial (RCT) evaluations of novel interventions which need highly skilled operators, and further problems to overcome in evaluations of surgery in particular [29]. These include the anxiety patients may have with leaving a decision between what can be quite different interventions to chance, ensuring a fair comparison between novel and standard techniques when surgeons are refining their skills in the former as the trial progresses, and keeping the patient and other assessors of outcome blind to individual treatment allocations, at least for the initial assessments of recovery. There is no doubt that patients undergoing esophagectomy are subject to a ‘complex intervention’, commonly defined as an intervention comprising multiple interacting aspects [30]. It is increasingly accepted that evaluations of such complex interventions should be preceded by feasibility work to establish if a main trial can recruit sufficient participants within an acceptable time, to standardize the interventions and establish criteria for their adequate implementation, and to establish the most appropriate measures of outcome for the main trial.

This protocol paper describes the ROMIO (Randomized Oesophagectomy: Minimally Invasive or Open) study in which we aim to standardize minimally invasive procedures for esophagectomy in order to refine our approach to evaluating MIO compared to open procedures in a RCT, and to determine the feasibility of a multicenter trial comparing the clinical and cost-effectiveness of minimally invasive and open surgical procedures in patients with cancer of the esophagus. The specific objectives of the ROMIO feasibility study are listed below.

### List of ROMIO feasibility study objectives

(i) pilot the randomization process and investigate difficulties affecting recruitment;

(ii) establish the proportion of patients who are potentially eligible and successfully recruited to inform sample size calculations for the main trial;

(iii) document in detail, using IDEAL recommendations[31], the technical developments of totally MIO to inform the design and choice of interventions in the main trial;

(iv) develop a manual for optimizing pathology procedures and reporting, including lymph node counts and ascertainment of positive resection margins, which are likely to be short-term outcome measures for the main trial;

(v) consider the appropriate statistical model for estimating treatment effectiveness;

(vi) develop and evaluate feasible, acceptable, and effective methods of blinding patients for the first week postoperatively, so reducing bias in self-reported outcomes; and

(vii) establish outcome measures for the main trial that enable a comprehensive, valid, and reliable assessment of esophagectomy outcome, and which include a set of core outcome measures considered to be essential in clinical effectiveness RCTs of esophageal cancer surgery.

## Methods/Design

ROMIO is a two-year external feasibility study with a pilot parallel group RCT at its core. The pilot trial is recruiting at two UK centers: University Hospitals Bristol NHS Foundation Trust and Plymouth Hospitals NHS Trust. Both centers have a team of upper gastrointestinal cancer surgeons (six in Bristol and five in Plymouth) and each undertake 50 to 100 resections for esophago-gastric cancer per year.

### Participants

**Figure 1:**
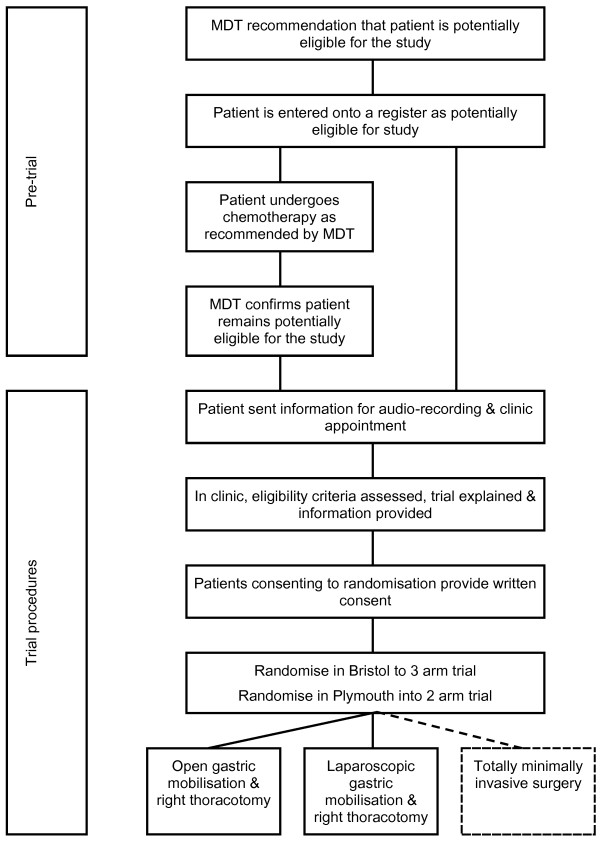
**Flow diagram showing process of recruitment to the ROMIO pilot randomized controlled trial.** MDT, multidisciplinary team.

All referrals of patients with esophageal cancer or high-grade dysplasia requiring surgery for primary esophagectomy or neoadjuvant chemotherapy before esophagectomy at the Bristol and Plymouth centers are considered for eligibility. Patients recommended for neoadjuvant chemotherapy and surgery are registered in the screening log but full eligibility is only confirmed once chemotherapy is completed, restaging undertaken, and the multidisciplinary team (MDT) of clinicians confirms that they will proceed to surgery. At this point eligible patients are informed about the trial and invited to have the method of their esophagectomy randomly selected from between open gastric mobilization and right thoracotomy, laparoscopic gastric mobilization and right thoracotomy, or (Bristol center only) totally minimally invasive surgery (Figure 1).

### Inclusion criteria

Participants may enter the study if ALL of the following apply: (1) male or female patients, (2) over 18 years of age, (3) referred by the MDT for primary esophagectomy or esophagectomy following restaging after neoadjuvant chemotherapy (of any type including chemoradiotherapy), (4) confirmed histopathological evidence of esophageal or esophago-gastric junctional adenocarcinoma, squamous cell cancer, or high-grade dysplasia, (5) fit for preoperative anaesthesia and surgery, assessed by the MDT, (6) able to provide written informed consent, (7) endoscopic measurement before chemotherapy that the tumor starts more than 5 cm below the crico-pharyngeus, (8) endoscopic measurement before chemotherapy that the tumor involves less than 4 cm of the gastric wall, and (9) the final pretreatment tumor stage is between high-grade dysplasia and T4aN1M0.

### Exclusion criteria

Participants may not enter the study if any of the following apply: (1) stage 4 disease, (2) type three tumors of the esophago-gastric junction that are scheduled for total gastrectomy, (3) patients with squamous cell cancer of the esophagus who the MDT recommends for, or who individually elect to undergo, definitive chemoradiotherapy, (4) evidence of previous complex thoracotomies or laparotomies, (5) evidence of previous or concomitant malignancy that would interfere with this treatment protocol, (6) pregnancy, (7) patients participating in other trials that would interfere with the implementation of this protocol at a particular study center.

### Randomization

Internet-accessed randomization is conducted centrally at the Bristol Randomized Trials Collaboration (BRTC), a UK Clinical Research Collaboration registered Clinical Trials Unit hosted by the University of Bristol. Randomization within blocks of varying size is conducted separately for the two centers and further stratified by whether the patient has undergone neoadjuvant treatment or not. In Bristol, patients are randomly allocated in a 1:1:1 ratio to one of three arms: open esophagectomy (open gastric mobilization and right thoracotomy), laparoscopic-assisted esophagectomy (laparoscopic gastric mobilization and right thoracotomy) or totally minimally invasive surgery. In Plymouth, randomization is restricted to two of the study arms, open esophagectomy or laparoscopic-assisted esophagectomy (Figure 1), in a 1:1 ratio. Patients must be logged into the trial and issued with a unique study ID number prior to the treatment allocation being generated, so ensuring judgments about eligibility are made without knowledge of what the next allocation will be (allocation concealment).

### Trial interventions

During the trial the surgical procedures are carried out under general anaesthesia with all patients receiving antibiotic and deep vein thrombosis (DVT) prophylaxis according to local hospital policies. The surgical procedures last between 5 and 8 hours. For the purposes of this pragmatic trial each intervention is allowed to be implemented according to the standard local policy, so long as there is consistency with the aspects of each intervention the study team currently consider as mandatory or prohibited for the particular method, as described below. This approach will be formalized in a process evaluation during this feasibility study leading to the production of an intervention manual and an assessment of the adequate standard of, and fidelity to, each procedure undertaken. The process evaluation will consider the surgical intervention itself and concomitant interventions.

### Open esophagectomy

In the ROMIO feasibility study the operation consists of a two-phase esophagectomy (abdomen and right chest) with a two-field lymphadenectomy (abdomen and thorax) and involves two key steps or phases.

### Abdominal phase

The incision (midline or subcostal) is at the surgeon’s discretion. Complete gastric mobilization will be performed based on the right gastroepiploic and right gastric arteries. Pyloroplasty, pyloromyotomy, or no drainage is at the surgeon’s discretion. Lymphadenectomies are performed along the common hepatic artery and the left gastric and splenic artery either *en bloc* or separately, and removal of sufficient crural fibres and a cuff of diaphragm performed if required for tumor clearance. The pericardial fat pad and strips of pleura are removed. Transection of the lesser curve may be undertaken or left to the thoracic phase of the operation. Placement of a feeding jejunostomy or naso-jejunal tube is at the surgeon’s discretion as are placement of intra-abdominal and intra-thoracic drains. Methods to close the abdomen are at the surgeon’s discretion.

### Thoracic phase

The chest is opened through a right thoracotomy and the mediastinal pleura overlying the esophagus excised in continuity with the esophagus. The posterior limit of the dissection should be the antero-lateral wall of the aorta. The thoracic duct is mobilized *en bloc* or separately to the esophagus and periesophageal tissues. The thoracic duct is ligated and divided at the level of the diaphragm. The esophagus is mobilized to the level of at least the aortic arch or higher if required. The para-esophageal and diaphragmatic nodes are removed in continuity with the esophagus. The lymph nodes at the tracheal bifurcation and along the right and left main bronchi to the pulmonary hilus can be removed *en bloc* or separately at the surgeon’s discretion. The anastomotic technique and method of chest drainage is at the surgeon’s discretion. Methods to close the chest are at the surgeon’s discretion.

### Laparoscopically assisted esophagectomy

This operation consists of identical steps as described for the open esophagectomy, but access to the abdominal cavity is achieved with four or five 10 or 5 mm incisions and surgery performed laparoscopically. Placement of a feeding jejunostomy is at the surgeon’s discretion and may be performed laparoscopically or by extending a port site to an 8 cm abdominal incision. The thoracic part of the operation is performed as described for the open esophagectomy.

### Totally minimally invasive esophagectomy

This consists of performing the steps of the abdominal and chest phases of the operation as described for the open esophagectomy. These are performed with abdominal and chest ports positioned by the surgeon at their discretion. Within the ROMIO feasibility study it is also possible to perform a three phase procedure (undertaking the anastomosis with a left cervical incision). Surgeons in Bristol undertaking the totally minimally invasive procedure are recording variations to this outlined approach, with reasons, during this feasibility study to inform the design and interventions to be evaluated in the main trial.

### Concomitant interventions and the enhanced recovery protocol

Concomitant interventions are defined as naturally accompanying or associated elements of the surgical intervention itself, and can be divided into preoperative, perioperative and postoperative components. Concomitant interventions to be considered as part of the process evaluation during the pilot trial include the anaesthetic and other perioperative procedures, immediate postoperative care (including intensive care management), and patient rehabilitation, input from allied health professionals such as physiotherapists and dieticians, which may or may not be encompassed into a formal enhanced recovery program. During the ROMIO feasibility study standard protocols for follow-up care after both procedures will be developed to minimize the risk of performance bias arising from carers differentially providing co-interventions in the main trial.

This work will inform development of an enhanced recovery pathway or manual to be used in the main trial to provide the minimum standard of care permitted. Together with the surgical intervention itself, concomitant interventions will be considered during the process evaluation and incorporated into the manual if identified as important.

### Outcome measures

The primary outcome for the main trial is currently planned to be patient-reported physical fatigue, as measured by the Multidimensional Fatigue Inventory MFI-20 [32,33] measuring fatigue at several time points during the first three months post-surgery; the speed of recovery of each patient will be captured.

During the pilot trial we are asking participants to complete a range of measures which are being considered as secondary outcome measures for the main trial [34-36]. These are listed below. In addition, post-surgery morbidity will be classified according to both the Accordian and Clavien-Dindo schemes [37,38].

### List of pilot trial secondary outcome measures

Date and cause of death

Disease recurrence with date

Lung function

Patient completed visual analogue scale assessment of pain

Patient completed questionnaires on generic HRQL: EuroQoL EQ-5D-5 L

Patient completed questionnaires on disease-specific HRQL: European Organisation for Research and Treatment of Cancer Core Quality of Life Questionnaire (EORTC QLQ-C30) and the esophageal specific module (QLQ-OES18)

### Development of a core clinical outcome set for esophageal cancer surgery

The ROMIO feasibility study provides the opportunity to develop a core outcome set for esophageal cancer surgery [39,40]. A core outcome set is a standardized set of clinical outcomes that represents the minimum that should be measured and reported in all clinical trials [40]. A relatively long list of potential outcomes will be generated by surveying patients and clinicians, which will be reduced to those considered crucial and distinctive through a Delphi exercise. This work will link with 'COMET' (Core Outcome Measures in Effectiveness Trials), funded by the Medical Research Council ConDuCT (Collaboration and innovation in difficult randomized controlled trials) Hub for Trials Methodology Research and North West Hubs for trials methodology research [40]. The final core set of outcomes for esophageal cancer surgery is expected to be less than 10 items.

### Development of resource use data collection

During the ROMIO study best methods for capturing cost and resource use in relation to the interventions and follow-up in secondary care are being established. Questionnaires are also being developed in order to collect information about the use of primary NHS services and social services and direct and indirect costs incurred by patient and carers. The average costs for all the different categories will be compared by study arm to enable the main cost drivers of the interventions to be established and compared. Any areas where obtaining accurate estimates of costs is problematic will also be identified. This will allow a more focused collection of resource use data and a more accurate estimate of cost-effectiveness in the main trial.

### Data collection

Socioeconomic details and height are measured when the patient is seen for a pre-surgery assessment only. Lung function measurements and assessments of pain are taken pre-surgery, during the first week post-surgery, and at days three and six as a minimum. HRQL is assessed using standardized questionnaires pre-surgery and at 6, 42, 90, and 185 days; questionnaires are posted with a stamped addressed envelope when necessary. Resource use is assessed through medical records review and interviews with patients.

Through medical records review, routine clinical measures are also captured whenever they are taken as part of the patient’s care during the six month study period. Clinical report forms designed for the study are used to formalize this data collection. Measures of the surgical process (for example blood loss or duration of surgery) and pathological assessment of the tumor and lymph nodes are included in this collection.

### Sample size calculation and statistical analysis

In the feasibility study recruitment is occurring initially over a 12-month period, with 72 potentially eligible patients expected during that time. This will allow a true 50% recruitment rate to be estimated with a 95% confidence interval of approximately 38 to 62%. If 11 patients are randomly allocated to each surgical procedure this will allow a true difference of 1.25 standard deviations between two procedures on a continuous measure of early outcome to be detected with 80% power at the 5% significance level. Hence the pilot trial will provide an acceptably precise estimate of the recruitment rate to inform plans for the main trial, and may provide evidence suggesting that a particular method may be achieving relatively poorer short-term outcomes and may need to be refined before proceeding to the main study.

Summary statistics that will inform plans for the main trial will be presented, including (in a CONSORT chart format) the number of potentially eligible and confirmed patients per month per center and the percentage of patients agreeing to randomization and completing outcome measurements. Mean scores on short-term outcome measures will be presented for each study arm, with *P* values and 95% confidence intervals presented for treatment comparisons where at least 10 patients have been randomized to each study arm. No conclusions about the relative clinical effectiveness of the three interventions will be drawn from these results; they will purely inform the refinement of the interventions prior to proceeding to the main trial. Additional summary statistics will arise from the pilot work, for example, mean scores on the blinding scale achieved by different blinding procedures.

### Quality control of surgery and development of the surgical manual

Only surgeons or trainees under direct supervision perform the procedures. A sample of procedures is being video recorded for analysis by the research team at Imperial College London. This will inform the development of the following resources for the main trial: (1) a surgical manual to define the framework for the steps of each trial intervention and describe acceptable and prohibited (unacceptable) protocol deviations; (2) an esophageal competency-assessment tool (O-CAT) based on the Observational Clinical Human Reliability Assessment (OCHRA) techniques to assess the level of competency for technical surgical performance [41], including adherence to procedural steps, protocol deviations, errors, and near miss events; and (3) a manual to describe details of concomitant interventions, such as type of anaesthesia, pre- and postoperative rehabilitation, and key elements of enhanced recovery pathways which are important in fulfilling the CONSORT criteria for reporting evaluations of complex interventions [30].

Pathological specimens are processed in an agreed uniform manner in both centers and dissection of lymph nodes from the main specimen and lymphadenectomy specimens follow a pro forma. These processes are reviewed regularly in both centers and standardized techniques for sampling lymph nodes are adopted so that the maximum yield can be obtained from all cases. Involvement of the surgical resection margin is assessed both microscopically and macroscopically.

### Blinding patients

Methods to achieve blinding of patients and outcome assessors to the type of surgery during the initial post-surgery period are being piloted. In the first seven days post-surgery patients are blinded by using large adhesive dressings that are positioned similarly on all trial patients regardless of the type of surgery (covering the abdominal, thoracic, and cervical incisions). Patients are asked to turn their head away during dressing changes and on days two and six they are asked to complete the Bang Blinding Index, which assesses blinding success by asking patients to guess their arm allocation [42]. The Bang Blinding Index is administered by ward or nursing staff not routinely involved in the patient’s care. Patients’ experiences of blinding and experiences of ward staff and nurses involved with these processes are being further explored in qualitative interviews described below.

### Recruitment investigation

The ROMIO trial compares different surgical procedures that are in common use in specialist centers, and therefore the trial is likely to face a number of recruitment challenges. Based on previous works by de Salis *et al.* and Donovan *et al.*[43-49], a key component of the pilot trial is an integrated qualitative study which is exploring these challenges to inform the design of the main ROMIO trial. Interviews with members of the trial management group, principal investigators and active recruiters explore their own views about the trial including their knowledge of the evidence and equipoise, and any recruitment challenges expected or experienced. The patient pathway is mapped through recruitment and appointments are audio recorded to scrutinize information provision and identify issues potentially affecting trial recruitment along that pathway. This will inform the development of information and training programs for the main trial. Finally, interviews with a sample of eligible patients will explore patient perspectives of surgery, previous experiences with treatments, views about surgery, and the acceptability of randomization between the procedures.

Emerging issues related to trial design and conduct that may be hindering recruitment are discussed between the trial team and a plan to improve recruitment during the pilot trial will be introduced if necessary. This may include reconsideration of eligibility criteria, study information, advice about presenting the study, discussions about equipoise or evidence, issues with patient pathways, and logistical issues in particular centers. These may be addressed by changes to study information or the protocol, or training for recruiters.

### Ethical approval and informed consent

Ethical approval was granted by the South West - Frenchay Research Ethics Committee (12/SW/0161). Informed consent is obtained from each participant in the ROMIO study.

## Discussion

Currently, there is a lack of published data from RCTs to support the patient benefit of minimally invasive surgery for esophageal cancer. The well-known challenges with conducting surgical trials need to be overcome to ensure that a multicenter pragmatic trial can be carried out efficiently. The ROMIO pilot trial has been designed as a necessary precursor to a main trial, during which the methodology and infrastructure for a trial will be established.

To have impact on clinical practice we anticipate that the main study will need to demonstrate more rapid recovery following MIO and the hybrid procedure compared to open esophagectomy, whilst showing that the same survival benefits are achieved. In the following illustrative calculations it is assumed that the between-center variation in outcome is accommodated by an intra cluster correlation of 0.1, and that each center will recruit 40 participants [50]. With the anticipated primary outcome measure of fatigue, the MFI-20 [32,33], 425 patients in each group would allow a difference of one half of a standard deviation to be demonstrated when comparing two treatment groups, with 90% power at the 5% significance level. Based on a one-sided 95% confidence interval, this sample size would also allow an absolute mortality risk difference at 12 months of 15% or more to be ruled out, with 80% power, when comparing the minimally invasive (22.5% mortality) to the open procedure (7.5% mortality).

The ROMIO pilot study has been designed to allow centers to opt to recruit into a two or a three arm study. Where the three arm trial has been performed, the technique for totally minimally invasive surgery has been deliberately allowed to evolve during the pilot study to document the technical changes and outcomes. Whether a totally minimally invasive approach can be used in many centers in the main trial is uncertain because it is not widely practiced in the UK. The final design of the main trial, therefore, will be considered by the trial oversight steering group in discussion with the trial management group and be selected to reflect current UK practice and emerging evidence.

## Trial status

The ROMIO feasibility trial recruited and randomized the first patient in April 2013. Recruitment is above the expected target and the feasibility work will be completed on schedule. The main trial is under design at present.

## Abbreviations

BRTC: Bristol randomized trials collaboration; COMET: Core outcome measures for effectiveness trials; EORTC: European organisation for research and treatment of cancer; HRQL: Health-related quality of life; HTA: Health technology assessment; MDT: Multidisciplinary team; MFI-20: Multidimensional fatigue inventory; MIO: Minimally invasive esophagectomy; NHS: National health service; RCT: Randomized controlled trial; ROMIO: Randomized oesophagectomy: minimally invasive or open; UHBT: University Hospitals Bristol NHS foundation trust.

## Competing interests

The authors declare that they have no competing interests.

## Authors’ contributions

KA drafted the manuscript based on the trial proposal and protocol, reviewed the protocol and developed the qualitative aspects. CPB designed the trial proposal and provided surgical expertise. RB designed the trial proposal and provided surgical expertise. JB drafted the manuscript based on the trial proposal and protocol, designed the trial proposal, provided surgical expertise, developed aspects of the protocol related to quality assurance of surgery and developed health economics aspects of the protocol. JD designed the trial proposal and developed qualitative aspects of the protocol. JE reviewed the protocol and contributed to aspects related to patient and public involvement. SF provided oncological expertise and reviewed the trial proposal. RG developed pathology aspects of the protocol. GH developed aspects of the protocol related to developing the manual and quality assurance of surgery. AH provided surgical expertise and designed the trial protocol. RK provided surgical expertise and reviewed the trial protocol. CM drafted the manuscript based on the trial proposal and protocol, designed the trial proposal and developed the statistical aspects of the protocol. SN developed health economics aspects of the protocol. CS provided surgical expertise and reviewed the trial protocol. GS provided surgical expertise and reviewed the trial protocol. DT provided surgical expertise and reviewed the trial protocol. TW provided surgical expertise and designed the trial protocol. All authors read and approved the final manuscript.
